# Ftr82 Is Critical for Vascular Patterning during Zebrafish Development

**DOI:** 10.3390/ijms18010156

**Published:** 2017-01-13

**Authors:** Hsueh-Wei Chang, Wen-Der Wang, Chien-Chih Chiu, Chiou-Hua Chen, Yi-Shan Wang, Zih-Ying Chen, Wangta Liu, Ming-Hong Tai, Zhi-Hong Wen, Chang-Yi Wu

**Affiliations:** 1 Department of Biological Sciences, National Sun Yat-sen University, Kaohsiung 80424, Taiwan; changhw@kmu.edu.tw (H.-W.C.); cchiu@kmu.edu.tw (C.-C.C.); z333134@hotmail.com (C.-H.C.); ki200412@yahoo.com.tw (Y.-S.W.); ying8215@gmail.com (Z.-Y.C.); 2 Institute of Medical Science and Technology, National Sun Yat-sen University, Kaohsiung 80424, Taiwan; 3 Department of Biomedical Science and Environmental Biology, Kaohsiung Medical University, Kaohsiung 80708, Taiwan; 4 Department of Medical Research, Kaohsiung Medical University Hospital, Kaohsiung 80708, Taiwan; 5 Department of Bioagricultural Science, National Chiayi University, Chiayi City 60004, Taiwan; wangw4@mail.ncyu.edu.tw; 6 Department of Biotechnology, Kaohsiung Medical University, Kaohsiung 80708, Taiwan; liuwangta@kmu.edu.tw; 7 Institute of Biomedical Science, National Sun Yat-sen University, Kaohsiung 80424, Taiwan; minghongtai@gmail.com; 8 Doctoral Degree Program in Marine Biotechnology, National Sun Yat-sen University and Academia Sinica, Kaohsiung 80424, Taiwan; wzh@mail.nsysu.edu.tw; 9 Department of Marine Biotechnology and Resources, National Sun Yat-sen University, Kaohsiung 80424, Taiwan

**Keywords:** *ftr82*, TRIM family, angiogenesis, ISV (intersegmental vessel), CVP (caudal vein plexus), zebrafish

## Abstract

Cellular components and signaling pathways are required for the proper growth of blood vessels. Here, we report for the first time that a teleost-specific gene *ftr82* (*finTRIM family*, member 82) plays a critical role in vasculature during zebrafish development. To date, there has been no description of tripartite motif proteins (TRIM) in vascular development, and the role of *ftr82* is unknown. In this study, we found that *ftr82* mRNA is expressed during the development of vessels, and loss of *ftr82* by morpholino (MO) knockdown impairs the growth of intersegmental vessels (ISV) and caudal vein plexus (CVP), suggesting that *ftr82* plays a critical role in promoting ISV and CVP growth. We showed the specificity of *ftr82* MO by analyzing *ftr82* expression products and expressing *ftr82* mRNA to rescue *ftr82* morphants. We further showed that the knockdown of *ftr82* reduced ISV cell numbers, suggesting that the growth impairment of vessels is likely due to a decrease of cell proliferation and migration, but not cell death. In addition, loss of *ftr82* affects the expression of vascular markers, which is consistent with the defect of vascular growth. Finally, we showed that *ftr82* likely interacts with vascular endothelial growth factor (VEGF) and Notch signaling. Together, we identify teleost-specific *ftr82* as a vascular gene that plays an important role for vascular development in zebrafish.

## 1. Introduction

The establishment of blood vessels is critical for embryonic development, and occurs in two distinct stages: vasculogenesis and angiogenesis. The de novo formation of new vessels through angioblast aggregation, migration, specification, and lumenization is termed vasculogenesis. Sprouting and growth of new vessels from preexisting vascular structures is termed angiogenesis. The regulation of vasculogenesis and angiogenesis is involved in multiple cell types, signaling molecules, and interaction pathways to coordinate the formation of the vascular system [1,2,3,4,5]. The zebrafish *Danio rerio* is an excellent model for the study of gene functions and signaling pathways in the field of vascular development because of the transparency of embryonic stages and many useful transgenic lines that express fluorescent tags in endothelial cells [6,7]. During zebrafish development, formation of the dorsal aorta and the posterior cardinal vein via the specification of angioblast precursor cells is considered vasculogenesis, and the subsequent sprouting and extension of the intersegmental vessels (ISVs) from the dorsal aorta or the process of caudal vein plexus (CVP) formation from the posterior cardinal vein is termed angiogenesis [8,9].

Specific genes controlling artery/vein specification of vasculogenesis have been identified in many vertebrate species [5,10]. In zebrafish embryos, sonic hedgehog (Shh) and vascular endothelial growth factor (VEGF) activate Notch signals via PLC-γ-PKC-MEK pathways and are required for arterial specification [11,12,13,14]. A distinct genetic program that activates PI3K/AKT signaling in angioblasts promotes venous specification [15,16,17]. However, the precise mechanism of signaling interactions in vascular development has yet to be elucidated. From the angiogenesis aspect, VEGF-Notch signaling also plays a central role in ISV patterning, including the specification, proliferation, and migration of endothelial cells [18,19]. Meanwhile, BMP (bone morphogenetic proteins) signaling regulates angiogenic sprouting from the posterior cardinal vein to form the caudal vein plexus (CVP), which is a distinct mechanism of angiogenesis from ISV growth [20,21,22]. Thus, a complex coordination among these signals and molecules has a critical role in controlling endothelial tip cell fates during angiogenesis. However, it is still not fully known which molecules are required for ISV and CVP formation. Identification of the regulators in these processes will enhance our understanding of the patterning of vascular networks, and could provide novel therapeutic targets.

In mammals, the tripartite motif proteins (TRIM) family are involved in various cellular processes, including cell growth, differentiation, and apoptosis [23,24,25]. The biological function of TRIM proteins in many studies is related to innate immunity against viral infection [24]. *ftr82* (*finTRIM family*, member 82) belongs to a large new subset family of tripartite motif proteins, called finTRIMs, which contain a RING-Bbox-coiled coil motif followed by different C-terminal domains. It was first described in teleost rainbow trout by virus induction [24]. FinTRIMs harbor nearly identical RING/B-box regions and C-termini with a B30.2 domain compared to TRIMs of fish and mammals. The zebrafish genome is very diverse, with 84 *finTRIM* genes on different chromosomes. A phylogenetic analysis showed the number of *fintrim* genes varies among fish species. The evolution and features of finTRIMs suggest an important role in the innate immunity of fish. The closest mammalian relatives are *trim16* and *trim25*, which have been shown to function in anti-viral infection, but are not true orthologs. It has been shown that *bloodthirsty* (*bty*) is an *RBCC/TRIM* gene required for erythropoiesis in zebrafish [26]. Other than these, there is limited information about TRIMs in fish studies, and no description of TRIM proteins related to vascular function is known.

In this study, we examine the function of *ftr82* in vascular development. We found that *ftr82* mRNA is expressed in developing vessels, and the loss of *ftr82* by morpholino (MO) knockdown impairs vascular patterning which reduces the expression of vascular markers. We further confirmed that *ftr82* MO knockdown works efficiently and is gene-specific. We next showed that the growth defect of endothelial cells in *ftr82* morphants is likely due to a decrease of cell proliferation and migration, but not the death of endothelial cells. Finally, we showed that *ftr82* likely interacts with VEGF and Notch signaling. Together, we identify teleost-specific *ftr82* as a vascular gene that plays an important role for the vascular development in zebrafish.

## 2. Results

### 2.1. The Expression of ftr82 Is in Developing Vessels

**Figure 1:**
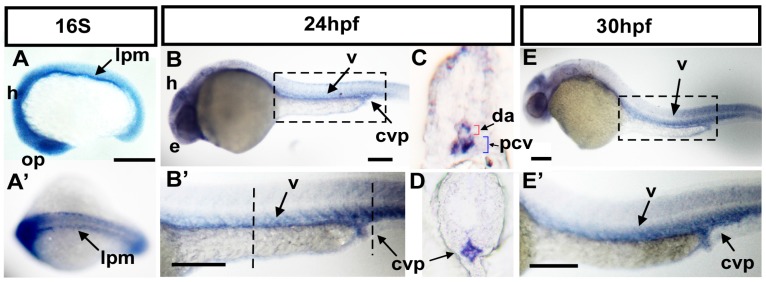
Expression pattern of *ftr82* mRNA during zebrafish development. (**A**) Lateral view of *ftr82* mRNA expression is observed at the 16 somite (16S) stage in the brain, optic placode (op), and lateral plate mesoderm (lpm); (**A’**) Dorsal view of embryos shows that *ftr82* is expressed in two bilateral stripes of presumptive angioblasts within lpm; (**B**,**B’**); At 24 h post-fertilization (hpf), *ftr82* is expressed in brain, eye (e) of the head, and in vessels (v) and caudal vein plexus (CVP) of the trunk; (**B’**) is an expanded image of **B**; (**C**,**D**) Transverse sections of embryo trunk and tail region from **B**’ show that *ftr82* is expressed in dorsal aorta (da), posterior cardinal vein (pcv), and CVP; (**E**,**E’**) At 30 hpf, *ftr82* expression continues in the head, vessels (v), and CVP of the trunk; (**E’**) is an expanded image of E. Scale bars are 200 µm.

We are interested in characterizing molecules required for vascular patterning [27,28,29]. Our unpublished transcriptome screening suggests that a teleost-specific *ftr82* gene is potentially involved in vascular development. To characterize the potential role of *ftr82* in the vascular development of zebrafish, we performed whole-mount in situ hybridization to examine the expression pattern of *ftr82* during zebrafish embryogenesis. At the 16 somite stage (S), *ftr82* is expressed in the optic placode (op), brain, head (h), and lateral plate mesoderm (lpm) (Figure 1A). A dorsal view of embryos showed that *ftr82* is also expressed at the lpm (Figure 1A’). The expression at the lpm corresponds to the location in the developing vessels. At 24 h post-fertilization (hpf), *ftr82* is expressed in the eye (e), brain, in the vessels (v), and in the caudal vein plexus (CVP) of the trunk (Figure 1B,B’). Transverse sections of embryos at 24 hpf further confirmed that *ftr82* is expressed in vessels, including dorsal aorta (da), posterior cardinal vein (pcv), and CVP (Figure 1C,D). At 30 hpf, *ftr82* expression continues in the head, trunk vessels, and CVP (Figure 1E,E’). The data show the expression of *ftr82* in developing vessels and suggest the function of *ftr82* in vascular development.

### 2.2. Knockdown of ftr82 Causes Vascular Defects during Zebrafish Development

**Figure 2:**
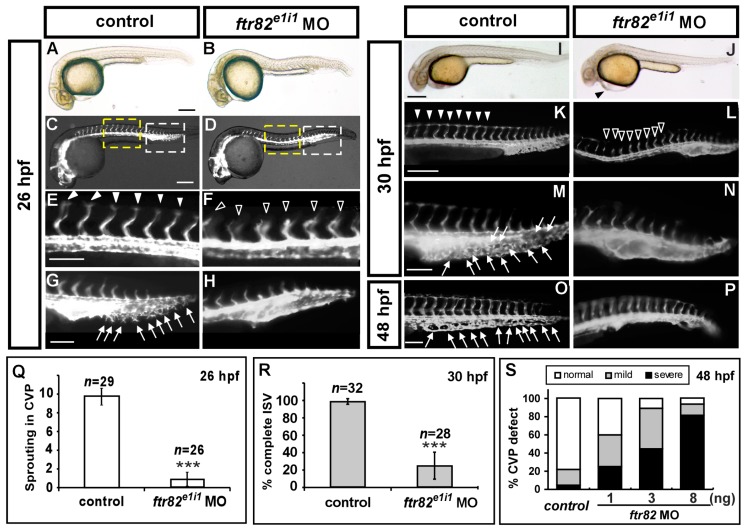

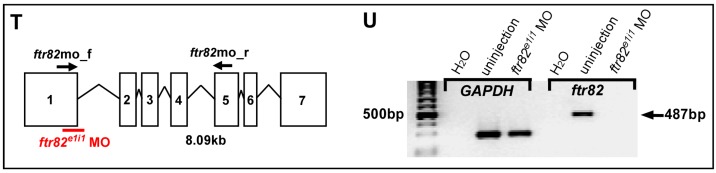
Knockdown of *ftr82* causes vascular defects by using splicing block morpholino. (**A**,**B**) Bright field images of uninjected control and *ftr82^e1i1^* morpholino injection at 26 hpf; (**C**–**H**) At 26 hpf, loss of *ftr82* shows intersegmental vessel (ISV) growth defect (hollow arrowheads in (**F**)) and less or no angiogenic sprouting from the caudal vein compared to uninjected controls (arrowheads in (**E**) and arrows in (**G**)) at 30 hpf; (**E**,**G**) are enlarged figures from (**C**), and (**F**,**H**) are enlarged figures from (**D**). At 30 hpf, in uninjected control embryos, ISV has reached the dorsal longitudinal anastomotic vessel (DLAV) at the dorsal aspect of the embryo (**K**, arrowheads), and the CVP formed honeycomb-like structures at the tail (**M**, arrows). At the same stage, ISVs are stalled at mid-somite in *ftr82^e1i1^* morphants (**L**, hollow arrowheads), and less honeycomb structure in the CVP (**N**); At 48 hpf, less or no CVP capillary loop can be observed (**P**) compared to the control (**O**, arrows); (**Q**) Quantification of angiogenic sprouting from the caudal vein shows a five-fold decrease in *ftr82* morphants at 26 hpf (*n* = 29 in control; *n* = 26 in *ftr82* MO); (**R**) Quantification of percentage of completed ISV shows a ~60% increase compared to *ftr82* morphants (*n* = 32 in control and *n* = 28 in *ftr82* MO) at 30 hpf; (**S**) Quantification of the deformation of CVP in *ftr82* morphants by different morpholino dosage injections (1, 3 and 8 ng) shows MO in a dose-dependent manner at 48 hpf; (**T**,**U**) The efficiency of *ftr82* splicing morpholino knockdown in embryos; (**T**) Schematic drawing showing the exon-intron structure of the *ftr82* gene, the targeting area of the *ftr82* splicing morpholino (*ftr82*^e1i1^ MO), and the suggested loss of the expression fragment can be detected by an *ftr82*mo_f and *ftr82*mo_r primer set; (**U**) cDNA from uninjected controls or 3.4 ng morpholino*-*injected *ftr82* morphants were subjected to PCR with primers for the loading control *GAPDH* or for the *ftr82* gene. In *ftr82*^e1i1^ morpholino-injected embryos, *GAPDH* levels at 283 bp are unchanged, while the amount of *ftr82* product at 487 bp is diminished, indicating the loss of *ftr82* expression caused by the morpholino inhibition. (*** refers to *p* < 0.0001 by an unpaired Student’s *t*-test.) Scale bars are 200 μm for **A**–**D**,**I**–**L** and 100 μm for **E**–**H**,**M**–**P**.

In mammals, the TRIM protein family are involved in various cellular processes, and in many studies, concerned with the innate immunity against viral infection. There is limited information about TRIM or finTRIM proteins related to vascular function in vertebrates to date. Meanwhile, the function of *ftr82* is still unknown in zebrafish. To identify the role of *ftr82* in vascular development, we use transgenic *Tg* (*kdrl:eGFP*)*^la116^* embryos that express green fluorescent protein (GFP) in endothelial cells. We knocked down *ftr82* expression by injecting 3.4 ng of splice-blocking morpholino that targeted the exon 1-intron 1 boundary (*ftr82^e1i1^* MO). Loss of *ftr82* showed two obvious vascular phenotypes: ISV growth defect and CVP mis-patterning (Figure 2C–H). At 26 and 30 hpf, a high percentage of ISV showed growth at the mid-somite, and did not reach the dorsal longitudinal anastomotic vessel (DLAV) (Figure 2D,F,K,L). There is only 20% of complete ISVs (*n* = 28 embryos) in *ftr82^e1i1^* morphants, compared to 80% of complete ISVs in uninjected controls (*n* = 32 embryos). We also observed defects in CVP formation as a second phenotype. At 26, 30, and 48 hpf, endothelial cells in the uninjected embryos’ CVP region showed angiogenic sprouting, migration, and the formation of honeycomb structures/loops (Figure 2G,M,O). Less or no sprouting was observed and quantitated at 26 hpf (Figure 2H,Q), and disruption of the honeycomb structure was observed in the CVP when compared to uninjected controls at 30 hpf (Figure 2M). At 48 hpf, a swollen plexus or fewer CVP capillary loops could be observed (Figure 2S). Quantification of CVP defects showed a dose-dependent decrease in *ftr82* morphants at 48 hpf. Thus, our data indicated that *ftr82* may play a critical role in controlling endothelial tip cell behaviors and in regulating ISV and CVP formation during angiogenesis. We further tested the efficiency of *ftr82^e1i1^* morpholino knockdown by real-time PCR (RT-PCR) analysis. We showed that the injection of 3.4 ng of *ftr82^e1i1^* morpholino diminished the normal fragment of *ftr82* (Figure 2U), indicating the loss of *ftr82* expression caused by morpholino inhibition.

### 2.3. Loss of ftr82 Showed Edema, Circulation Defect, and Absent Parachordal Chain

**Figure 3:**
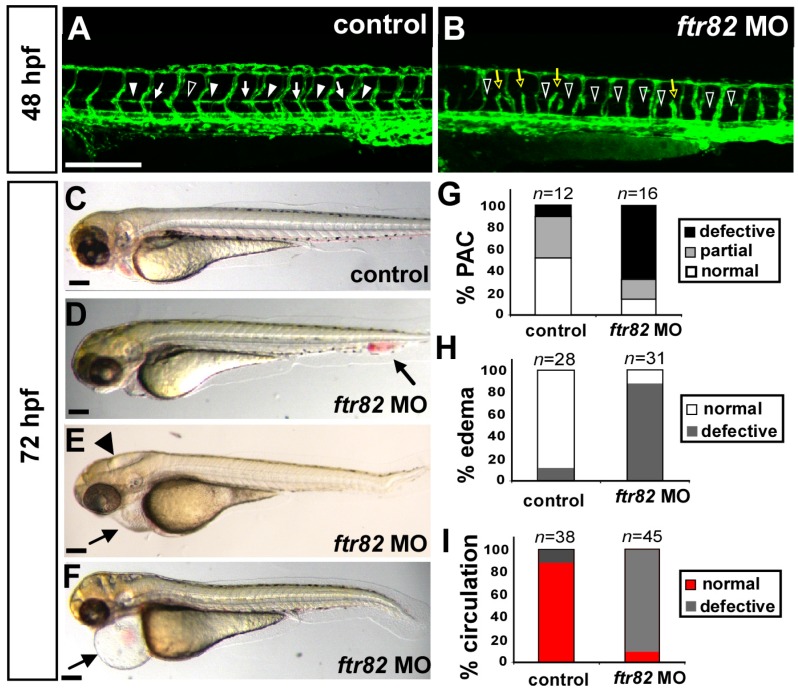
Loss of *ftr82* showed edema, circulation defect, and absent parachordal chain. (**A**,**B**) At 48 hpf, *ftr82* morphants showed incomplete ISV ((**B**), yellow hollow arrows) and absent parachordal chain (PAC) formation ((**B**), hollow arrowheads) compared to control (**A**) by using *Tg (fli1a:eGFP)^y1^* fish; (**G**) Quantification of PAC formation with normal (arrowheads in (**A**)), partial growth (arrows in (**A**)) or absence (hollow arrowhead in (**A**)); (**C**–**F**) At 72 hpf, representative embryos showed loss of *ftr82*, resulting in an increasing edema in the brain (arrowhead in (**E**)) and pericardium (arrows in (**E**,**F**)) or blood accumulation at CVP (arrow in (**D**)); (**H**) Quantification of *ftr82* morphants showed ~90% of embryos with circulation defects in the axial vessels and/or ISVs-DLAVs (*n* = 38 in control and *n* = 45 in *ftr82* MO); and (**I**) 80% of *ftr82* morphants (*n* = 31) with mild to severe edema and/or blood blockage compared to control (*n* = 28). The scale bars in (**A**,**B**) represent 200 μm and in (**C**–**F**) are 500 μm.

By using *Tg* (*fli1a:eGFP*)*^y1^* fish as endothelial specific promoters of fli1a-driven GFP allows for the examination of parachordal chain (PAC) formation. The PAC is a string of endothelial cells located at the horizontal myoseptum and forms at 48 hpf and PAC cells eventually migrate ventrally along the trunk vasculature to the space between the dorsal aorta and posterior cardinal vein to form the main vessel of the lymphatic system, the thoracic duct (TD). Loss of *ftr82* showed incomplete ISV (yellow hollow arrow in Figure 3B) and the absence of PAC formation (white hollow arrowhead in Figure 3B) when compared to the control. The defect of PAC formation was quantitated in the defective or partial growth in Figure 3G. At 72 hpf, we found that *ftr82* morphants showed increasing blood accumulation at CVP, edema at brain or pericardium regions (Figure 3C–F). Embryos showed ~80% edema at 72 hpf (*n* = 28 in control and *n* = 31 in *ftr82* MO) (Figure 3H). Blood accumulation at CVP is consistent with the defects of CVP and loss of circulation. While we examined the circulation in uninjected controls and morphants, we noted limited blood flow in the trunk of *ftr82* morphants at 48–72 hpf. *ftr82* morphants showed ~10% of embryos with circulation in the axial vessels and/or ISV circulation (*n* = 38 in control and *n* = 45 in *ftr82* MO) (Figure 3I). Since edema and the blockage of circulation are common secondary effects of vessel impairment, the data is consistent with the vasculature defects in the *ftr82* knockdown embryos.

### 2.4. Knockdown of ftr82 Can Be Rescued by Overexpression of ftr82

**Figure 4:**
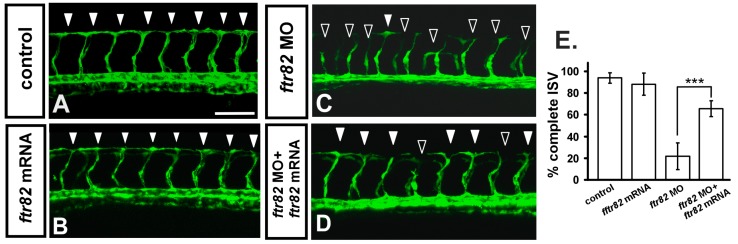
Knockdown of *ftr82* can be rescued by overexpression of *ftr82*. In uninjected control embryos, ISV has reached the dorsal region and formed DLAV around 28–30 hpf ((**A**), arrowheads). At the same stage in *fr82* MO, ISVs have stalled or slowed growth at mid-somite ((**C**), hollow arrowheads). Overexpression of *ftr82* by *ftr82* mRNA injection caused no obvious defect in vasculature (**B**), but rescued the defect of ISV stalling ((**D**), solid arrowheads); (**E**) Quantification of the percentage of completed ISV at 30 hpf shows a ~45% increase in rescued embryos compared to *ftr82* morphants. Percentages of completed ISV are ~97, 22 ± 12, 92 ± 8, and 67 ± 7 in control, *ftr82* MO, *ftr82* mRNA overexpression, and rescued embryos, respectively. *** refers to *p* < 0.0001 by an unpaired Student’s *t*-test. Data represent means ± S.D. Scale bars are 100 μm for (**A**–**D**).

We showed that the loss of *ftr82* causes vascular defects by splice-blocking morpholino knockdown (Figure 2). To test that the knockdown of *ftr82* is being targeted specifically, we first tested the efficiency of *ftr82^e1i1^* morpholino knockdown by analyzing *ftr82* expression products (Figure 2U). To further test phenotypic specificity of the *ftr82* morpholino knockdown, we tested the effects of an additional morpholino targeting the translation initiation site (*ftr82^atg^* MO). Our results showed similar vascular defects in *ftr82^atg^* MO (Figure S1) when compared to *ftr82^e1i1^* morphants. We showed vascular defects in CVP sprouting at 26 hpf (four-fold decrease; Figure S1E,F,O), ISV growth at 30 hpf (50% decrease; Figure S1G,H,P), and ISV cell number at 30 hpf (1.9-fold decrease; *n* = 22 in controls and *n* = 25 in *ftr82* morphants; Figure S1K,L,Q). The vascular defect at CVP capillary loop formation is also observed at 48 hpf (Figure S1M,N). To confirm the vascular defects caused by morpholino knockdown specifically, we performed rescue experiments by overexpression of *ftr82* in control and *ftr82^e1i1^* morphant embryos. At 30 hpf, we found that overexpression of *ftr82* mRNA in *ftr82* MO restores ISV growth to form DLAV by 45% compared to injection of *ftr82* morpholino alone (*ftr82* MO) (Figure 4C–E), while overexpression of *ftr82* in control embryos has no obvious effect on the vascular development (Figure 4B) when compared to uninjected controls (Figure 4A). The data suggest that knockdown of *ftr82* indeed impaired vascular development. In addition, we have assessed the gross developmental process which is unaffected in *ftr82* morphants, because knockdown of *ftr82* did not alter the expression of heart, gut, somite, and neural systems (Figure S2). Meanwhile, loss of *ftr82* did not cause developmental delay by measuring the heart rate at 24–25 hpf (Figure S3).

### 2.5. Knockdown of ftr82 Impairs the Growth of ISV Cells

**Figure 5:**
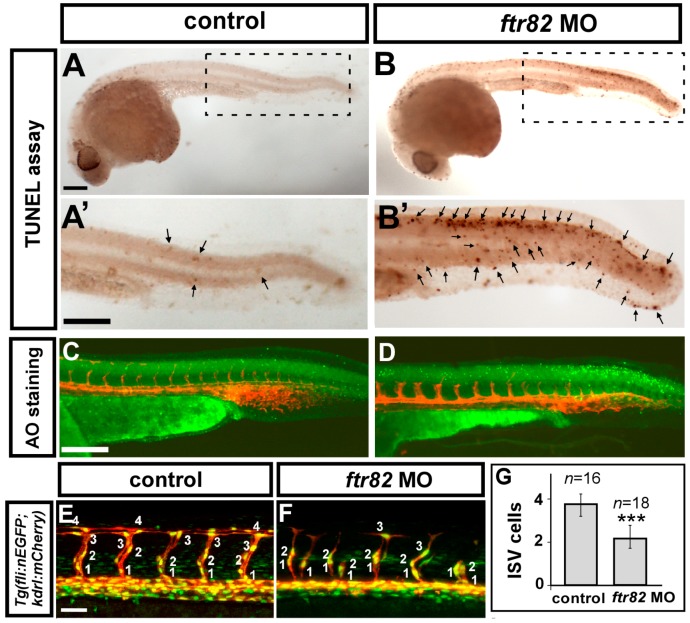

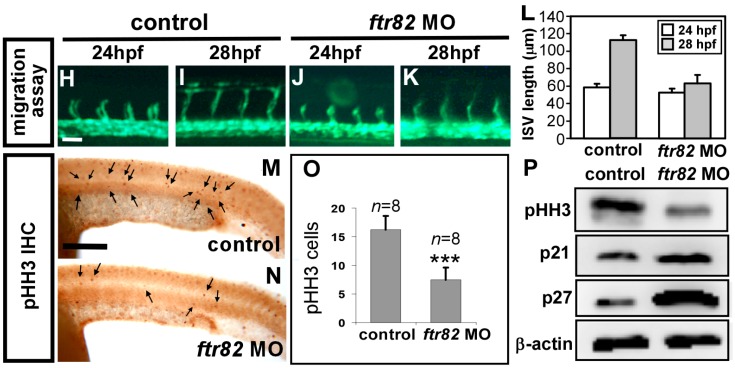
Loss of *ftr82* impairs the growth of ISV cells. (**A**,**A’**,**B**,**B’**) TdT-mediated dUTP-X nick end labeling (TUNEL) assay was used to detect apoptotic cells in uninjected control and *ftr82^e1i1^* morphants. Increased apoptotic cells were observed on the skin and in the epidermis of the dorsal tail region (arrows), but not in vascular regions in *ftr82* MO compared to controls at 28 hpf; (**A’**,**B’**) are expanded images of (**A**) and (**B**), respectively; (**C**,**D**) AO staining (green dots) in *Tg* (*kdrl:mCherry*) fish exhibited more apoptotic cells with the knockdown of *ftr82*; (**E**,**F**) The number of cells forming each ISV counted in control *Tg* (*kdrl:mCherry^ci5^*; *fli1a:negfp ^y7^*) (**E**) and *ftr82* morphant embryos (**F**) at 32 hpf; (**G**) Quantification of average ISV cells per ISV counted in both control (*n* = 16) and *ftr82* morphant (*n* = 18); (**H**–**L**) migration assay measured the difference of ISV length from 24 to 28 hpf in control and *ftr82* MO (*n* = 30 ISVs from three control embryos or morphants); (**M**,**N**) Proliferation marker pHH3 was counted in the trunk region beneath the neural tube and above the yolk extension area, where it is more related to main vessels and ISVs; (**O**) The mean number of mitotic cells (phosphohistone H3, pHH3 cells) in control was 16.3 ± 2.4 (*n* = 8) and in *ftr82* MO was 7.5 ± 2.1 (*n* = 8); (**P**) Western blot analysis showed the reduced expression level of pHH3; the increase of p21 and p27 protein levels. β-actin serves as a loading control. Scale bars are 200 μm for (**A**–**D**,**A’**,**B’**,**M**,**N**) and 50 μm for (**E**,**F**,**H**–**K**). *** refers to *p* < 0.0001 by an unpaired Student’s *t*-test.

Knockdown of *ftr82* results in vascular growth defects, suggesting the interruption of endothelial cell migration and/or proliferation, or an increase of cell death. To test these possibilities, we first performed a TdT-mediated dUTP-X nick end labeling (TUNEL) assay and acridine orange (AO) staining to detect apoptotic cells. We showed that *ftr82* morphants increase apoptotic cells at the epidermis of the dorsal tail region compared to uninjected embryos; however, morpholino-induced cell death has no significant increase in the vessel region of trunk (Figure 5A–D,A’–B’), suggesting that the vascular defect is not due to the endothelial cell death. To test if loss of *ftr82* would affect cell proliferation, we analyzed the numbers of endothelial cells per ISV in the *Tg* (*kdrl:mCherry^ci5^; fli1a:negfp ^y7^*) embryos, where GFP was expressed in the nuclear of endothelial cells and the mCherry tag on endothelial ISV cells. Loss of *ftr82* showed reduced ISV cells significantly when compared to the uninjected control (Figure 5E–G, *n* = 18 in *ftr82* morphants and *n* = 16 in control, *p* < 0.0001). In addition, only a small portion of ISV cells can reach to the top of embryo to form DLAV, suggesting that endothelial cell migration is impaired in *ftr82* morphants. To confirm the migration defect, we measured the difference of ISV length from 24 hpf to 28 hpf in control and *ftr82* MO. The difference of ISV length in *ftr82* MO is significantly shorter than control, suggesting that the migration ability is impaired (Figure 5H–L). We further demonstrated the proliferation defect in *ftr82* MO by examining the expression levels of proliferation marker phosphohistone H3 (pHH3) and cell cycle negative regulators p21 and p27. Immunostaining with anti-phosphohistone H3 antibody recognizes mitotic cells, and the amount of mitotic cells in the trunk of *ftr82* MO showed less brown spots compared to control (arrows in Figure 5M,N). Western blotting also showed reduced pHH3 signal in *ftr82* MO. In addition, the increase of the expression of cell cycle inhibitors p21 and p27 suggested the impairment of cell proliferation. Together, the data suggest that *ftr82* is important for endothelial cell growth to pattern ISV and/or CVP, likely via regulation of the proliferation and/or migration of the cells.

### 2.6. Knockdown of ftr82 Affects the Expression of Vascular Markers

**Figure 6:**
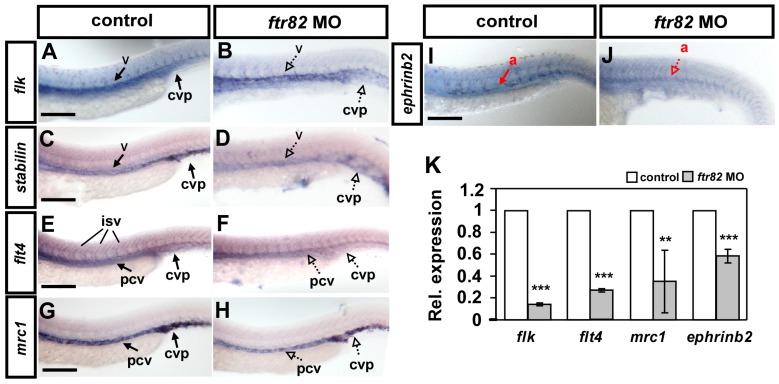
Knockdown of *ftr82* affects the expression of vascular markers. Compared to uninjected controls (**A**,**C**,**E**,**G**,**I**), the expression of the pan-vascular markers *flk* and *stabilin* at vessels (v) and caudal vein plexus (CVP) were decreased in *ftr82* morphants at 24 hpf (**A**–**D**). In addition, the expression of venous markers *flt4* (**E**,**F**) and *mrc1* (**G**,**H**) at posterior cardinal vein (pcv) and arterial marker *ephrinb2* at aorta (a), were reduced in *ftr82* morphants (**I**,**J**). (**K**) Quantification of the relative expression level by qPCR assay showed reduced expression in vascular markers *flk* (0.14 ± 0.01), *flt4* (0.26 ± 0.02), *mrc1* (0.35 ± 0.28), and *ephrinb2* (0.58 ± 0.06) in *ftr82* morphants. *** refers to *p* < 0.0001 and ** refers to *p* < 0.001 by an unpaired Student’s *t*-test. Data are represented as means ± S.D. Scale bars are 200 μm in **A**–**H**.

The vascular defects in ISV growth and CVP formation suggest that *ftr82* is critical for vascular development, and likely modulates vascular identity. To test this hypothesis, we examined the expression of several typical vascular markers, *flk*, *flt4*, *mrc1*, *stabilin*, and *ephrinb2*, at 24 hpf by whole-mount in situ hybridization. We found that the expression of the pan-vascular markers *flk* and *stabilin*, venous/ISV specific marker *flt4*, venous marker *mrc1*, and arterial marker *ephrinb2* were all decreased in *ftr82* morphants (dashed arrow in Figure 6B,D,F,H,J) compared to the uninjected controls. To determine the extent of vascular marker expression, we quantified *flk*, *flt4*, *mrc1*, and *ephrinb2* transcript levels by quantitative PCR (qPCR) analysis and identified a 40%–80% decrease in expression in *ftr82* morphants (Figure 6K). These results suggest that *ftr82* regulates several vascular genes to control vessel development.

### 2.7. Regulation between ftr82 and VEGFR2-Notch Signaling

**Figure 7:**
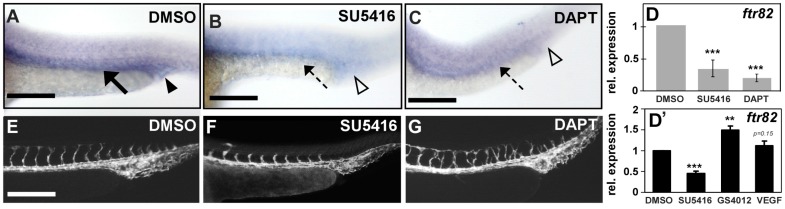
Regulation between *ftr82* and VEGFR2/Notch. (**A**,**B**) *ftr82* expression in vessels and CVP is reduced in VEGFR2-specific inhibitor SU5416 (10 μM)-treated embryos ((**B**), dashed arrow and hollow arrowhead) as compared to DMSO control embryos ((**A**), arrow and arrowhead); (**A**,**C**) *ftr82* expression is down-regulated after treatment with Notch inactivation chemical DAPT (75 μM) ((**C**), dashed arrow and hollow arrowhead) as compared to DMSO control embryos (**A**); (**D**) Quantification of the relative expression level by qPCR assay showed the reduced expression of *ftr82* in SU5416-treated (0.3 ± 0.18) or DAPT-treated (0.2 ± 0.1) embryos; (**D’**) qPCR assay showed the relative expression of *ftr82* in SU5416-treated (0.5 ± 0.1), GS4012-treated (1.5 ± 0.1), or VEGF-treated (1.1 ± 0.1) embryos; (**E**–**G**) Embryos treated with 10 μM SU5416 or 75 μM DAPT showed ISV growth defects and ISV mispatterns, respectively, but vascular structure did not disappear. *** refers to *p* < 0.0001 and ** refers to *p* < 0.001 by an unpaired Student's *t*-test. Data are represented as means ± S.D. Scale bars represent 200 μm in (**A**–**C**,**E**–**G**).

We showed that knockdown of *ftr82* by morpholino injection impaired ISV growth with a decrease of ISV endothelial cells. In addition, loss of *ftr82* causes the CVP defects of sprouting and capillary network formation. Notch signaling has been shown to be important for vein identity and the interaction of ISV tip cell with VEGFR2 signaling during angiogenesis. Thus, we tested the regulatory relationships between *ftr82* and Notch or VEGFR2. We inactivated Notch and VEGFR2 signaling by DAPT and SU5416 treatment, respectively. We found that *ftr82* expression was downregulated in vessels and CVP when Notch or VEGFR2 signals were inhibited by whole-mount in situ hybridization and qPCR analysis (Figure 7A–D). On the other hand, we induced VEGF signal by adding a pro-angiogenic compound GS4012 or VEGF in fish medium at 6 hpf, and found that the increase of *ftr82* expression though VEGF-treatment was not statistically significant (Figure 7D’). Although SU5416 or DAPT interrupt the growth of ISV and CVP, the main vascular structure does not disappear (Figure 7E–G), suggesting that the down-regulation of *ftr82* in drug treatment is not due to the loss of vasculature. Thus, *ftr82* plays an important role in vascular development, likely mediated by the Notch/VEGF pathways.

## 3. Discussion

In this study, we examined the function of *ftr82* in vascular development. We found that *ftr82* mRNA is expressed in developing vessels (Figure 1), and the loss of *ftr82* by morpholino knockdown impairs the growth of ISV and CVP (Figure 2), reduces circulation, impairs PAC formation (Figure 3), and changes the expression of vascular markers (Figure 6). We further showed that the *ftr82* MO knockdown works efficiently and functionally (Figure 2, Figure 4 and S1). Next, we demonstrated that the growth defect of endothelial cells in *ftr82* morphants is due to a decrease in cell proliferation and migration, but not cell death (Figure 5). However, without a test of individual endothelial cell migration and analysis of molecular markers, our data suggest that *ftr82* more likely modulates the proliferation of endothelial cells rather than their migration. Finally, we showed that *ftr82* likely interacts with VEGF and Notch signaling (Figure 7).

TRIM proteins contain a tripartite motif, a RING zinc finger, one or two B-boxes, and a coiled-coil domain. They have been shown as novel players in antiviral defense. Many members of this family contain E3 ubiquitin ligases activity that transfers the ubiquitin to a substrate protein, and this activity is RING domain dependent at N-termini [30]. Phylogenetic analyses suggest that finTRIM proteins appeared and diversified during the teleost evolution, while *TRIM25* and *TRIM39 (bty)* are more ancient genes common to all vertebrates [25]. The sharing of B30.2 domains suggests a putative target-binding region between two major protein families involved in immune recognition of pathogen-specific motifs and expects the role of finTRIMs in antiviral innate immune system [24]. Our future work will be aimed at a further understanding of the biological functions of Ftr82, in addition to its vascular function; for example, to test if Ftr82 has a function in antiviral immunity. Interestingly, from our observation in this study, we found that the loss of *ftr82* causes the defect of parachordal chain (PAC) formation in the lymphogenesis system at an early stage (Figure 3). In this aspect, *ftr82* contributes to the establishment of the lymphatic network for immune defense in addition to innate response of its potential antiviral defense. Meanwhile, the molecular mechanisms that *ftr82* control vascular patterning are unknown. Since Ftr82 shares the coiled-coil domain and B30.2 domains for protein–protein interaction, there might be other protein targets associated with the regulation of vascular patterning. This will be interesting to address in the future.

Alternatively, the RING finger domain of Ftr82 can serve as a putative interaction site with E2 ubiquitin-conjugating enzymes, and acts as E3 ubiquitin ligases. It will be interesting to test if Ftr82 shows E3 ubiquitin ligase activity. If yes, what is its substrate target? There are a couple of examples suggesting that E3 ubiquitin ligase functions in the regulation of vascular growth, such as Mib1 [31], Lnx1 [32], Fbxw7 [33], and Atrogin-1 [34]. Mindbomb homolog1 (Mib1) promotes endocytosis of the Notch ligands delta and Jagged. Loss of *mib1* causes overgrowth of vessels due to the impairment of tip-stalk cell identity [31]. The E3 ubiquitin ligase Fbxw7 has been shown responsible for the polyubiquitination and proteasomal degradation of substrates such as Notch and c-Myc. Izumi et al. showed that Fbxw7 controls angiogenesis by degradation of active Notch activity in a mouse retina and a zebrafish embryonic trunk [33]. In addition to the regulation of Notch signaling by E3 ubiquitin ligase, VEGF signaling can be regulated by E3 ubiquitin ligase, such as Ftr82 in this study.

## 4. Materials and Methods

### 4.1. Zebrafish Line, Maintenance, and Chemicals Treatment

Zebrafish (*Danio rerio*) were raised and maintained in a 28.5 °C environment as previously described [28,35,36]. The regulations are approved by the National Sun Yat-sen University Animal Care Committee (approval reference #10231). Wild-type AB or TL fish and transgenic lines *Tg* (*flk:GFP*)*^la116^*, *Tg* (*kdrl:mCherry*)*^ci5^*, and *Tg* (*fli1a:negfp*)*^y7^* [37,38,39] were obtained from the Taiwan Zebrafish Core Facility at Academia Sinica. To prevent pigment formation, embryos were treated with 0.003% 1-Phenyl-2-Thiourea (PTU, Sigma, St. Louis, MO, USA) at six hours post-fertilization (hpf). DAPT (*N*-[(3,5-Difluorophenyl)acetyl]-l-alanyl-2-phenylglycine-1,1-dimethylethyl ester, Calbiochem, Nottingham, UK), SU5416 (Calbiochem), GS4012 (Calbiochem) or VEGF (Sigma) chemical treatment is for the modification of signaling pathways. A stock solution of 25 mM DAPT, 10 mM SU5416, 20 mg/mL GS4012, and 10 mM VEGF dissolved in dimethyl sulfoxide (DMSO) were diluted to the working concentration and added to embryos fish medium at 6 hpf. Control embryos were treated with 0.3% of DMSO.

### 4.2. Embryo Morpholino and mRNA Injection

Morpholinos (MOs) were obtained from Gene-Tools (Philomath, OR, USA). Morpholinos are designed to block translation by targeting the first codon of *ftr82* (*ftr82^atg^* MO) or to disrupt splicing of *ftr82* (*ftr82^e1i1^* MO) by targeting the exon1-intron1 boundary and the sequences as follows: *ftr82^atg^* MO sequence: 5′-TGGAGACATTTGCTCAGCCATGTTT-3′; *ftr82^e1i1^* MO sequence: 5′-GCGCTATGTTTTCCTTACCTGTTTT-3′; the morpholinos were injected with 2 and 3.4 ng, respectively. Unless specified, all images show the phenotype of *ftr82 morphants* (*ftr82* MO) with 3.4 ng injection of splicing morpholino (*ftr82^e1i1^* MO). For rescue experiments, capped and polyadenylated mRNA of *ftr82* was synthesized in vitro using the mMESSAGE mMASCHINE kit (Ambion, Austin, TX, USA). All injections were performed into one-cell zebrafish embryos by using a FemtoJet microinjector (Eppendorf AG, Hamburg, Germany).

### 4.3. RNA Extraction, cDNA Preparation, and Quantitative Real-Time RT-PCR (qPCR) Analysis

Total RNA from certain stages of embryos were prepared using the RNeasy kit (QIAGEN, Valencia, CA, USA) according to the manufacturer’s instructions. One microgram of total RNA was used to generate cDNA using a mix of oligo-dT primer (Roche Applied Science, Branford, CT, USA) and the reverse transcriptase (RTase, Roche) according to the manufacturer’s instructions. Real-time PCR was performed using the LightCycle 96 real-time PCR detection system and SYBR Green I Master (Roche). Real-time RT-PCR primers are listed in Table S1. Relative cDNA amounts were calculated using the LC96 program and normalized to the expression of β-actin based on the ΔΔ*C*_t_ method. All reactions were performed as biological triplicates.

### 4.4. Morpholino Efficiency

The efficiency of morpholinos that cause mis-splicing or reduced band signals was determined using polymerase chain reaction (PCR) with primers franking on exon1 and exon 5 (Figure 2T) RNAs extracted from splicing morpholinos injected and uninjected embryos and reverse transcript to cDNA. Primers *ftr82*mo_f: 5′-TCCCTGTGACTTCTGCACTG-3′ and *ftr82*mo_r: 5′-CCTCTGACCTCATCCTCTCG-3′ were used to examine *ftr82* knockdown efficiency, while primers GAPDH_f: 5′-TGCTGTAACCGAACTCATTGTC-3′ and GAPDH_r: 5′-CAAGCTTACTGGTATGGCCTTC-3′ were used as a loading control.

### 4.5. Whole-Mount In Situ Hybridization and Cryosection

Whole-mount in situ hybridization was followed by the high-resolution protocol based on [40,41]. An antisense in situ probe for *ftr82* was obtained by PCR using primers *ftr82*_f: 5′-TGCAGCCGTACTCTGACAAC-3′ and *ftr82*_rT7: 5′-TAATACGACTCACTATAGTGTCCATCAATCATGCCTTC-3′ and in vitro transcription using T7 Polymerase (Roche) with digoxigenin-labeled UTP. The *flk*, *stabilin*, *flt4*, *mrc1*, and *ephrinb2* probes have been described [11,42]. Briefly, embryos were fixed using 4% paraformaldehyde (PFA) and stored at −20 °C in methanol. After rehydration and permeabilization, the probes were added to embryos in hybridization buffer at 65 °C overnight, then washed and blocking with 1% bovine serum albumin (BSA). After incubation with AP-conjugated anti-Dig antibody, they were washed, and NBT/BCIP substrate (Roche) was added for color development. Embryos were then embedded in 3% methylcellulose for photographing. For cryosection, embryos were fixed with tissue-freezing medium Tek O.C.T (optimum cutting temperature) compound and sectioned at 10 µm using a Leica CM3050S cryostat and photographed on an IX71 inverted microscope (Olympus, Tokyo, Japan).

### 4.6. Imaging and Photo Processing

Embryos were mounted either in 3% methyl cellulose (Sigma) or 1.5% low melt agarose (Invitrogen, Philadelphia, PA, USA). Images were photographed with an AxioCam high-resolution camera and processed by AxioVision software (Carl Zeiss, Jena, Germany). For the confocal images, embryos were immobilized and embedded in 1.5% low-melting-point agarose with 5% tricaine (Invitrogen), and images were collected on a Zeiss LSM700 or Nikon Eclipse 90i C1 confocal microscope and processed with ImageJ software (NIH, Bethesda, MD, USA). Final figures were made using Adobe Photoshop.

### 4.7. TUNEL Assay

Embryos were fixed with PFA and stored in methanol. Embryos were rehydrated, digested with 10 µg/mL Proteinase K (Roche), and fixed again in 4% PFA for apoptotic cell detection by using a TdT-mediated dUTP-X nick end labeling (TUNEL) assay kit (Roche). Briefly, fixed embryos were treated with 3% hydrogen peroxide for one h at room temperature and washed to eliminate endogenous peroxidase. Then, 45 µL TUNEL label solution was mixed with 5 µL of TUNEL enzyme and added into the embryos for three hours at 37 °C. After reaction, embryos were washed to remove unincorporated nucleotides and blocked with 5% horse serum, then incubated with peroxidase (POD) conjugated anti-fluorescein antibody (Roche) overnight at 4 °C, washed four times in PBT, and visualized using a 3,3′-diaminobenzidine (DAB) colormetric kit (Roche).

### 4.8. Acridine Orange Staining

The dechorionated embryos were soaked in E3 fish medium containing 2 µg/mL acridine orange (Sigma) for one hour. After washing six times with fresh E3 medium, the embryos were mounted in 3% methylcellulose with 5% tricaine and photographed.

### 4.9. Protein Extraction and Western Blotting

Embryo lysates were prepared using RIPA lysis buffer (50 mM Tris-HCl pH 7.4, 1% NP-40, 0.25% sodium deoxycholate, 150 mM NaCl, 1 mM phenylmethylsulfonyl fluoride (PMSF), and protease inhibitors) and phosSTOP (Roche). The lysates were spun down, added with SDS sample buffer, and boiled for 10 min. The proteins were separated by SDS-PAGE and then transferred to a polyvinylidene fluoride (PVDF) membrane (BioRad, Hercules, CA, USA). The membranes were washed in TBS (Tris-buffered saline) with Tween-20 (TBST) and blocked with nonfat milk in TBST for 1 h. The membranes were then incubated with anti-phospho histone H3 (pHH3) (Millipore, Billerica, MA, USA), anti-p21, anti-p27 (Santa Cruz Biotechnology, Santa Cruz, CA, USA) polyclonal antibodies, and anti-β-actin monoclonal antibody (Sigma) overnight at 4 °C. The membranes were washed with TBST and incubated with a peroxidase conjugated secondary antibody (Cell Signaling Technology, Danvers, MA, USA) at room temperature for 2 h. The signal was detected by ECL chemiluminescence (Bio-Rad).

### 4.10. Anti-Phospho Histone H3 Staining

Embryos were fixed with PFA, permeabilized with pre-chilled acetone for 10 min, washed in phosphate-buffered saline (PBS) with 0.1% Tween-20 (PBSTw), and blocked with 10% normal goat serum (NGS) in PBS with 0.8% Triton X-100 (PBSTx) for 1 h at room temperature. Then, embryos were incubated overnight at 4 °C in the anti-phospho histone H3 polyclonal antibody (Millipore; 1:300 in 1% NGS/PBSTx). After washing four times for 10 min each in PBSTw, the embryos were incubated overnight with the secondary antibodies (anti-rabbit IgG peroxidase conjugated, 1:300 in 1% NGS/PBSTx). Embryos were washed five times and incubated with DAB peroxidase substrate (Roche). The color reaction was stopped by washing with PBSTw, fixed for 30 min in 4% PFA, washed in PBSTw, and cleared in 70% glycerol/PBSTw.

## 5. Conclusions

In summary, we identify teleost-specific *ftr82* as a vascular gene that plays an important role for vascular development in zebrafish. We also showed that *ftr82* likely interacts with VEGF and Notch signaling to control vascular patterning. It will be interesting to address how the molecular function of *ftr82* acts in the future. Understanding this mechanism can provide the evolutionary difference of vascular development and immunity systems between mammals and fish.

## Supplementary Materials

Supplementary materials can be found at www.mdpi.com/1422-0067/18/1/156/s1.
