# Supplementary material for: Ftr82 Is Critical for Vascular Patterning during Zebrafish Development
Source: Int J Mol Sci. 2017 Jan 13;18(1):156. doi: 10.3390/ijms18010156 (PMC5297789; doi:10.3390/ijms18010156)
Supplement: Supplementary file 1 [file ijms-18-00156-s001.pdf]

Supplementary Materials: Ftr82 Is Critical for Vascular Patterning during Zebrafish Development

Hsueh-Wei Chang, Wen-Der Wang, Chien-Chih Chiu, Chiou-Hua Chen, Yi-Shan Wang, Zih-Ying Chen, Wangta Liu, Ming-Hong Tai, Zhi-Hong Wen and Chang-Yi Wu

Table S1. Quantitative PCR (qPCR) primer sequences used in this study.

qPCR Primers	Sequence
*β-actin*_qf	5′-CTCTTCCAGCCTTCCTTCCT-3′
*β-actin*_qr	5′-CTTCTGCATACGGTCAGCAA-3′
*flt4*_f1	5′-ACTCGGGTTATTACCGCTGCTTCT-3′
*flt4*_r1	5′-TGGATGCTCTGGGTCTCGAACAAA-3′
*flk1*_qf	5′-ACTTTGAGTGGGAGTTTCATAAGGA-3′
*flk1*_qr	5′-TTGGACCGGTGTGGTGCTA-3′
*mrc1*_qf	5′-CTAGCAAGCCTGAAGGTGCC-3′
*mrc1*_qr	5′-TGAGAGGCTGGGTAGTTGGG-3′
*ephrinb2*_qf	5′-CTGGAACACCACGAACACC-3′
*ephrinb2*_qr	5′-CACACGTGGGCAAACTATGT-3′
*ftr82*_qf	5′-TCCCTGTGACTTCTGCACTG-3′
*ftr82*_qr	5′-CCTCTTGAAGGTGGCAGACT-3′

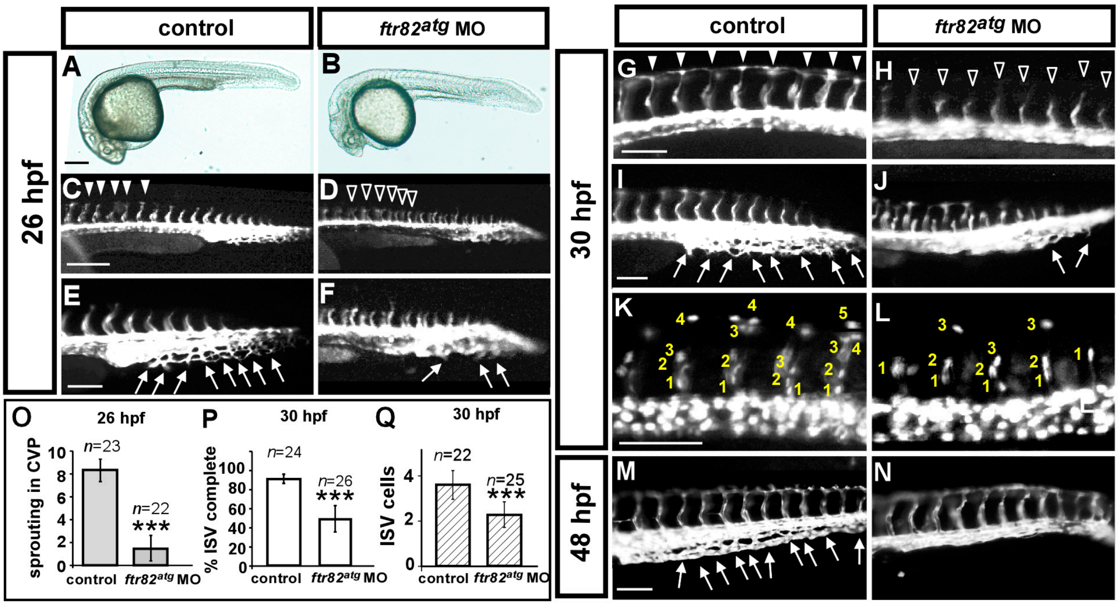

Figure S1. Knockdown of *ftr82* causes defects in vascular development in zebrafish by using translational block ATG morpholino. (**A**–**F**) Bright-field images show the morphology of uninjected control *Tg*(*flk:gfp*) embryos (control, **A**) and *ftr82* translational block ATG morpholino injection (*ftr82*^{atg} MO) at 26 hpf (**B**); (**C**–**F**) Loss of *ftr82* shows intersegmental vessel (ISV) growth defect (hollow arrowheads in (**D**)) compared to control (arrowheads in (**C**)), and less angiogenic sprouting from the tail caudal vein in *ftr82* morphants (arrows in **E**,**F**) at 26 hours post-fertilization (hpf). (**E**) and (**F**) are enlarged figures from (**C**) and (**D**), respectively; (**G**–**L**) At 30 hpf, in uninjected control embryos, ISV have reached the dorsal longitudinal anastomotic vessel (DLAV) at the dorsal part of the embryo (**G**, arrowheads) and the caudal vein plexus (CVP) formed loop structures at the tail (**I**, arrows). Knockdown of *ftr82* at the same stage showed ISVs growth stall in the middle line of somite (**H**, hollow arrowheads) and less honeycomb structure in the CVP (**J**, arrows); Reduced number of cells per ISV was photographed and counted in *ftr82* morphant embryos (2.0 ± 0.5, n = 25) versus in control *Tg*(*fli1a:negfp*)^{y7} (3.8 ± 0.4, n = 22) (**K**,**L**,**Q**). At 48 hpf, less or no capillaries loop in the CVP was observed (**N**) compared to control (arrows in **M**); (**O**) Quantification of angiogenic sprouting from the caudal vein shows a 4.5-fold decreased in *ftr82* morphants at 26 hpf (n = 23 in control and in n = 22 in *ftr82* MO); (**P**) Quantification of percentage of completed ISV in *ftr82* morphants shows a ~40% decrease (n = 24 in control and n = 26 in *ftr82*^{atg} MO) at 30 hpf. *** refers to $p < 0.0001$ by an unpaired Student's t-test. Scale bars are 200 μm for **A**–**D**, 100 μm for **E**–**N**.

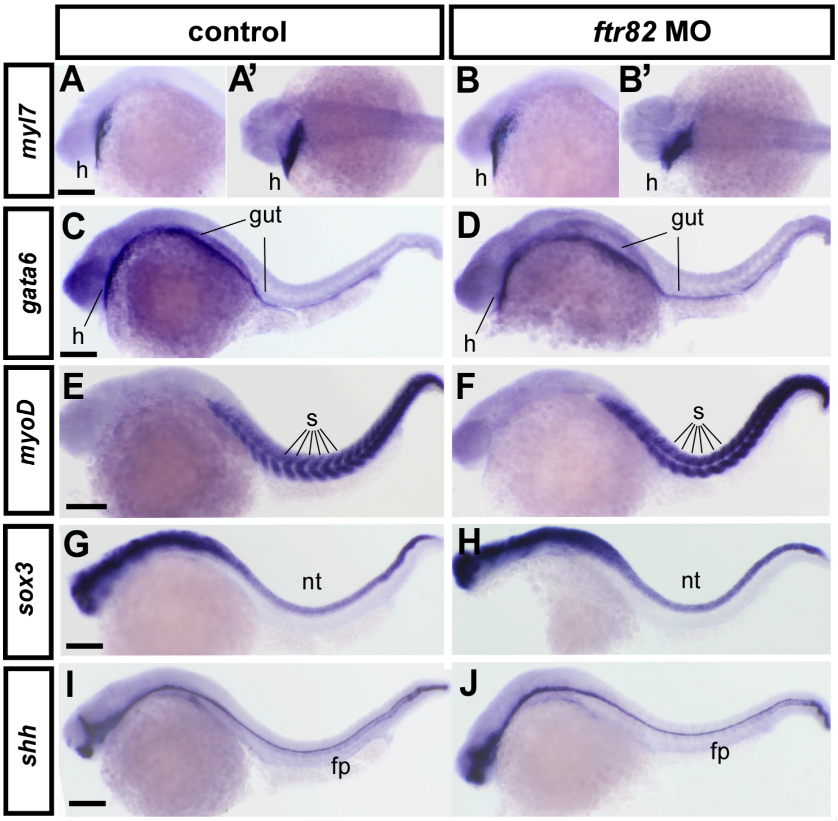

Figure S2. The gross developmental process is unaffected in *ftr82* morphants. Knockdown of *ftr82* at 24 hpf did not alter the expression pattern of heart, gut, somite, or neural systems by examining the expression of *myl7* (heart marker, h, **A** and **B** are lateral view and **A'** and **B'** are dorsal view), *gata6* (heart and gut marker, **C**,**D**), *myoD* (somite marker, s, **E**,**F**), and *sox3* (neural tube marker, nt, **G**,**H**) and *shh* (floor plate marker, fp, **I**,**J**). Scale bars in all figures are 250 μm.

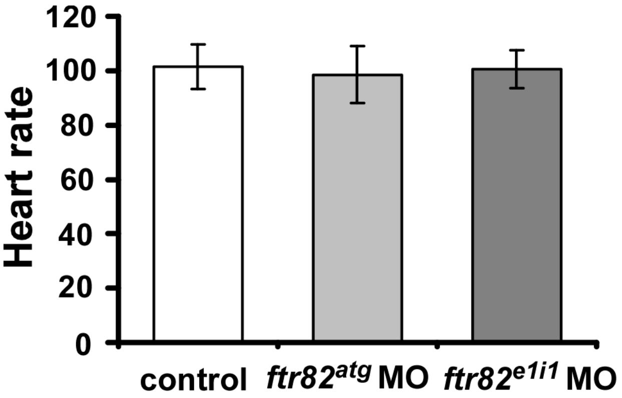

Figure S3. Knockdown of *ftr82* did not cause developmental delay by measuring heart rate. Quantification of zebrafish heart beats per minute in uninjected control and *ftr82* morphants shows no difference (n = 12 in control, n = 12 in $ftr82^{atg}$ MO and n = 9 in $ftr82^{e1i1}$ MO) at 24–25 hpf. The mean heart rate determined by direct visual examination of ventricle beating in control was 101.5 ± 8.2 beats per minute, 98.5 ± 10.5 beats per minute in $ftr82^{atg}$ MO, and 100.6 ± 6.9 beats per minute in $ftr82^{e1i1}$ MO.
